# A Candidate HIV/AIDS Vaccine (MVA-B) Lacking Vaccinia Virus Gene *C6L* Enhances Memory HIV-1-Specific T-Cell Responses

**DOI:** 10.1371/journal.pone.0024244

**Published:** 2011-08-31

**Authors:** Juan García-Arriaza, José Luis Nájera, Carmen E. Gómez, Nolawit Tewabe, Carlos Oscar S. Sorzano, Thierry Calandra, Thierry Roger, Mariano Esteban

**Affiliations:** 1 Department of Molecular and Cellular Biology, Centro Nacional de Biotecnología, Consejo Superior de Investigaciones Científicas (CSIC), Madrid, Spain; 2 Biocomputing Unit, Centro Nacional de Biotecnología, Consejo Superior de Investigaciones Científicas (CSIC), Madrid, Spain; 3 Infectious Diseases Service, Department of Medicine, Centre Hospitalier Universitaire Vaudois and University of Lausanne, Lausanne, Switzerland; The University of Chicago, United States of America

## Abstract

The vaccinia virus (VACV) C6 protein has sequence similarities with the poxvirus family Pox_A46, involved in regulation of host immune responses, but its role is unknown. Here, we have characterized the C6 protein and its effects in virus replication, innate immune sensing and immunogenicity *in vivo*. C6 is a 18.2 kDa protein, which is expressed early during virus infection and localizes to the cytoplasm of infected cells. Deletion of the *C6L* gene from the poxvirus vector MVA-B expressing HIV-1 Env, Gag, Pol and Nef antigens from clade B (MVA-B ΔC6L) had no effect on virus growth kinetics; therefore C6 protein is not essential for virus replication. The innate immune signals elicited by MVA-B ΔC6L in human macrophages and monocyte-derived dendritic cells (moDCs) are characterized by the up-regulation of the expression of IFN-β and IFN-α/β-inducible genes. In a DNA prime/MVA boost immunization protocol in mice, flow cytometry analysis revealed that MVA-B ΔC6L enhanced the magnitude and polyfunctionality of the HIV-1-specific CD4^+^ and CD8^+^ T-cell memory immune responses, with most of the HIV-1 responses mediated by the CD8^+^ T-cell compartment with an effector phenotype. Significantly, while MVA-B induced preferentially Env- and Gag-specific CD8^+^ T-cell responses, MVA-B ΔC6L induced more Gag-Pol-Nef-specific CD8^+^ T-cell responses. Furthermore, MVA-B ΔC6L enhanced the levels of antibodies against Env in comparison with MVA-B. These findings revealed that C6 can be considered as an immunomodulator and that deleting *C6L* gene in MVA-B confers an immunological benefit by enhancing IFN-β-dependent responses and increasing the magnitude and quality of the T-cell memory immune responses to HIV-1 antigens. Our observations are relevant for the improvement of MVA vectors as HIV-1 vaccines.

## Introduction

Poxvirus vectors express numerous genes encoding for immunomodulatory proteins that interfere with host anti-viral response [1]. The VACV *C6L* gene is present in the genome of VACV strains Western Reserve (WR) (*VACV-WR_022*), Copenhagen (*C6L*) and MVA (*MVA 019L*), but absent in New York Vaccinia Virus strain (NYVAC). *C6L* is presumably an immediate-early gene based on the analysis of the *C6L* promoter (www.poxvirus.org) and a genome-wide transcriptome analysis that detected C6 mRNA 30 minutes post-infection [2]. *C6L* encodes a 157 amino acid protein with a predicted molecular weight of 18.2 kDa (www.poxvirus.org). Bioinformatic analyses clustered *C6L* to the poxvirus BCL-2-like gene family that includes *A46R*, *A52R*, *B15R* (named *B14R* in WR) and *K7R* [3], a family of proteins that inhibit the Toll-like receptor (TLR) signalling pathway at different levels [4], [5], [6], [7], [8], [9], [10], [11], [12]. C6 protein is present at low levels in VACV intracellular mature virions (IMV) [13], and binds to KRT4 (keratin 4), PDCD6IP (programmed cell death 6 interacting protein) and TNNI2 (troponin I) [14]. Moreover, a C6 epitope (amino acids 74–82, GFIRSLQTI in WR and SFIRSLQNI in MVA) is highly immunogenic in BALB/c mice, and WR elicited high levels of *C6L*-specific IFN-γ secreting cells in mice, similarly to VACV *E3L*, *F2L* and *A52R* peptides [15]. All these characteristics suggest that C6 may have an important immunomodulatory function by antagonizing with the TLR signalling pathway.

The highly attenuated VACV strain MVA is one of the most promising vectors to be used as an effective vaccine against HIV-1 [16]. MVA has an excellent safety profile, and MVA recombinants expressing HIV-1 antigens induce protection after simian/human immunodeficiency virus (SHIV) challenge, and elicit strong, broad, polyfunctional and durable immune responses to HIV-1 antigens in different animal models and humans trials [[17], [18], [19], [20], [21], [22], for a review [23]].

We have previously constructed a recombinant MVA expressing codon-optimized Env as monomeric gp120 and the polyprotein Gag-Pol-Nef of HIV-1 from clade B (referred as MVA-B), that in DNA prime/MVA boost protocols in mice induced strong immune responses to HIV-1 antigens [17], [18], [20]. In macaques, a similar MVA construct expressing Env (gp120 from SHIV_89.6P_) and Gag-Pol-Nef (from SIV_mac239_) showed strong specific CD4^+^ and CD8^+^ T-cell immune responses with a bias for CD8^+^, and high protection after challenge with SHIV_89.6P_ [22]. Furthermore, the expression of HIV-1 antigens from MVA-B selectively induced in human dendritic cells (DCs) the expression of different cellular genes that might act as regulators of immune responses to HIV-1 antigens [24] and MVA-B-infected DCs co-cultured with autologous T lymphocytes induced a highly functional HIV-1-specific CD8^+^ T-cell responses including proliferation, secretion of IFN-γ, IL-2, TNF-α, MIP1β, MIP1α, RANTES and IL-6, and strong cytotoxic activity against autologous HIV-1-infected CD4^+^ T lymphocytes [25]. Based on these previous results, MVA-B has recently entered a phase I clinical trial in healthy volunteers in Spain. However, more efficient poxvirus MVA-B vectors that enhance the magnitude, breath, polyfunctionality and durability of the immune responses to HIV-1 antigens are desirable. This is particularly relevant when a single immunogen is desirable for mass vaccination purposes to simplify the immunization protocols and reduce manufacturing cost. Deletion in the vector backbone of MVA-B of known or suggested immunomodulatory VACV genes, which antagonize host specific immune responses, is a general strategy that could enhance immunogenicity of the vector against HIV-1 antigens.

In this study, we have generated a new HIV-1 vaccine candidate, termed MVA-B ΔC6L, which contains a deletion in the vector backbone of MVA-B of the VACV *C6L* gene. We have analyzed the expression of C6, the innate immune responses elicited by MVA-B ΔC6L in human THP-1 cells and monocyte-derived DCs (moDCs) and we have examined if the deletion of *C6L* gene in the candidate HIV/AIDS vaccine vector MVA-B could improve the humoral and T-cell memory immune responses to HIV-1 antigens in mice. We showed that C6 is expressed early during viral infection and localizes in the cytoplasm of infected cells. MVA-B ΔC6L replicates in cell culture at the same level as parental MVA-B, indicating that C6 is not essential for virus replication. Furthermore, MVA-B ΔC6L up-regulated the expression of IFN-β and IFN-α/β-inducible genes (IFIT1 and IFIT2) in human THP-1 cells and moDCs, suggesting that C6 inhibits the IFN-β signalling pathway by blocking some unknown component involved in the induction of IFN-β. In DNA prime/MVA boost immunization protocols in mice comparing MVA-B ΔC6L and MVA-B, MVA-B ΔC6L significantly enhanced the magnitude and polyfuncionality of the HIV-1-specific CD4^+^ and CD8^+^ T-cell memory immune responses, which are mostly mediated by CD8^+^ T cells of effector phenotype in both immunization groups. HIV-1-specific CD4^+^ T-cell memory responses induced by MVA-B and MVA-B ΔC6L were preferentially Env-specific. However, while MVA-B elicited more Env- and Gag-specific CD8^+^ T-cell memory responses, MVA-B ΔC6L induced preferentially Gag-Pol-Nef (GPN)-specific CD8^+^ T-cell memory responses. Moreover, MVA-B ΔC6L enhanced the levels of antibodies against HIV-1 Env in comparison to MVA-B.

Altogether, our findings revealed that C6 is a new VACV immunomodulatory protein, and that deletion of *C6L* in MVA represents an attractive alternative to increase the immunogenicity of MVA vaccine candidates.

## Results

### Generation and *in vitro* characterization of MVA-B ΔC6L

**Figure 1:**
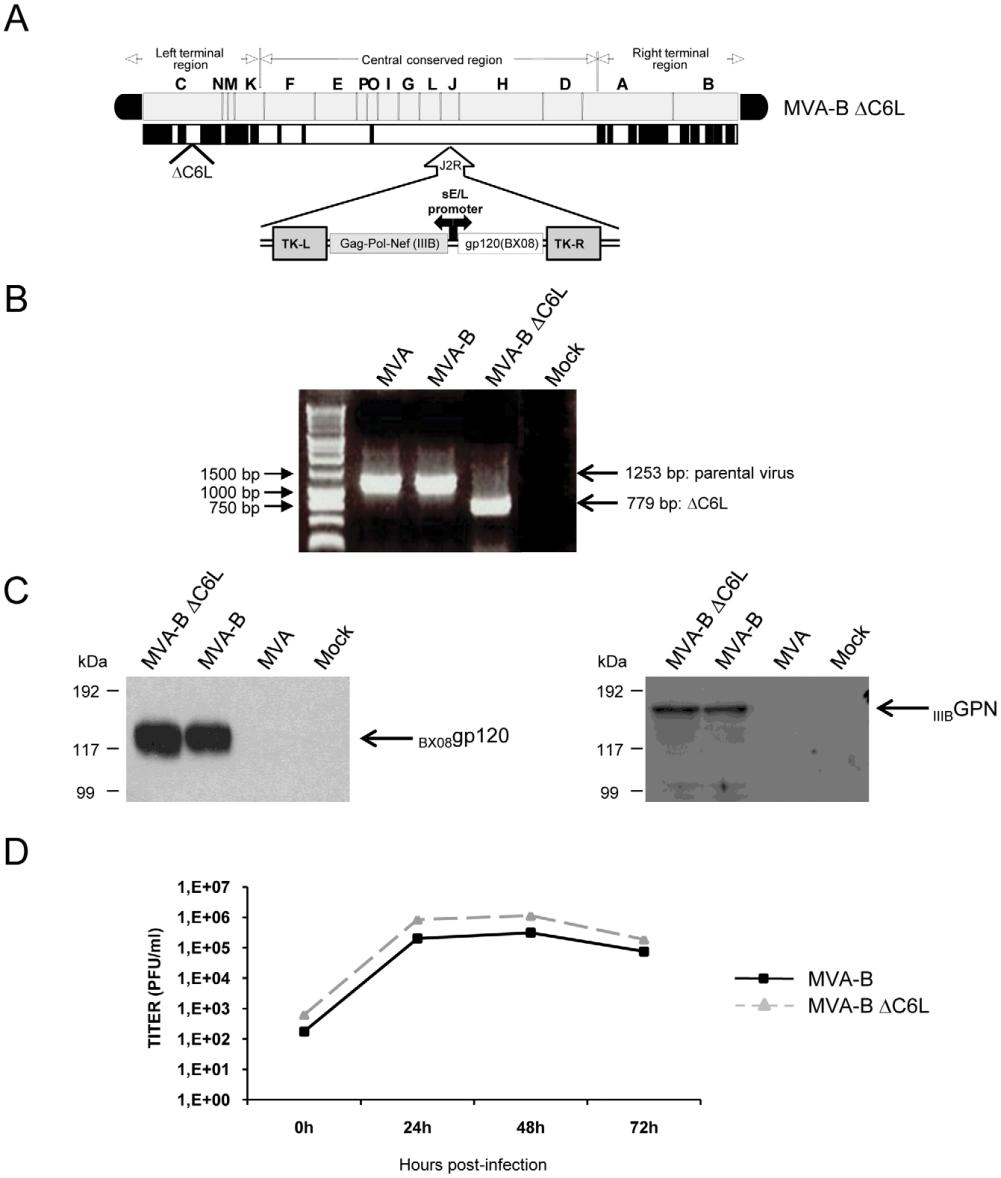
*In vitro* characterization of MVA-B ΔC6L deletion mutant. (A) Scheme of MVA-B ΔC6L genome map, adapted from [72] and [73]. The different regions are indicated by capital letters. The right and left terminal regions are shown. Below the map, the deleted or fragmented genes are depicted as black boxes. The deleted *C6L* gene is indicated. The HIV-1 Gag-Pol-Nef (from isolate IIIB) and gp120 (from isolate BX08) clade B sequences driven by the synthetic early/late (sE/L) virus promoter inserted within the TK viral locus (J2R) are indicated (adapted from [18]). (B) PCR analysis of *C6L* locus. DNA extracted from DF-1 cells infected at 2 PFU/cell with MVA, MVA-B or MVA-B ΔC6L was used for PCR analysis. The DNA products corresponding to the parental virus or to the deletion are indicated by an arrow on the right, with the expected size in base pairs. Molecular size marker (1 Kb ladder) with the corresponding sizes (base pairs) is indicated on the left. Lane Mock, cells not infected. (C) Expression of HIV-1 _BX08_gp120 and _IIIB_GPN proteins in DF-1 cells infected (2 PFU/cell) with MVA-B and MVA-B ΔC6L, at 24 h post-infection. (D) Virus growth of MVA-B and MVA-B ΔC6L in infected (0.01 PFU/cell) DF-1 cells at different times and titrated by plaque immunostaining assay with anti-WR antibodies. The mean of three independent experiments is shown.

The function of the VACV *C6L* is unknown, although it is predicted that have an immunomodulatory role [3]. To analyze the possible immunomodulatory function of *C6L*, we have constructed an MVA-B deletion mutant lacking VACV gene *C6L* (MVA-B ΔC6L, see *Materials and Methods*) from the previously described recombinant MVA-B (expressing HIV-1 Env, Gag, Pol and Nef antigens from clade B) [18]. The diagram of the MVA-B ΔC6L deletion mutant is shown in Figure 1A. PCR using primers for the *C6L* locus confirmed the deletion of the *C6L* gene in MVA-B ΔC6L (Figure 1B). Deletion of *C6L* from MVA-B ΔC6L was also confirmed by DNA sequencing (data not shown). In addition, analysis by Western blot confirmed that MVA-B ΔC6L expresses HIV-1 antigens _BX08_gp120 and _IIIB_GPN at the same level as their parental virus MVA-B (Figure 1C).

### C6 is non-essential in cell culture

The mere isolation of MVA-B ΔC6L deletion mutant demonstrated that the C6 protein is not essential for MVA replication. To determine whether deletion of *C6L* altered virus multiplication, we compared the growth of MVA-B ΔC6L and MVA-B in DF-1 cells. Kinetics studies revealed that deletion of *C6L* in the MVA-B genome did not affect virus replication. Hence, *C6L* is not essential for virus propagation in cultured cells (Figure 1D). Furthermore, similar to the parental virus MVA-B or MVA, MVA-B ΔC6L is an attenuated virus which does not replicate in mammalian cells (Figure S1).

### C6 is expressed early in infection

**Figure 2:**
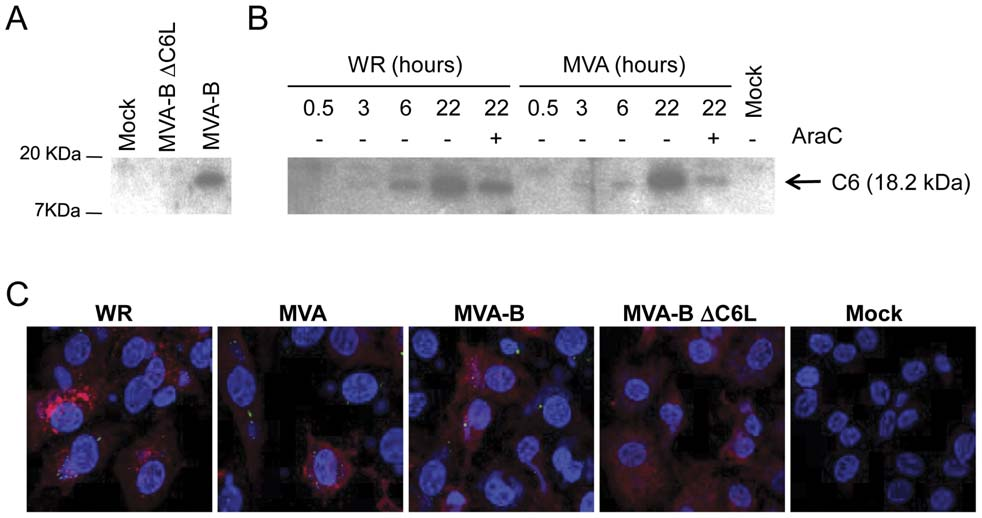
Characterization of C6 expression and localization. DF-1 cells were infected with 5 PFU/cell of MVA-B and MVA-B ΔC6L (A) or WR and MVA (B) in the presence or absence of AraC (B). Cells extracts collected 24 hours post-infection (A) or at the indicated time (B) were analyzed by SDS-PAGE. VACV C6 protein was detected by Western blot using rabbit polyclonal sera against C6. (C) DF-1 cells were infected with WR, MVA, MVA-B or MVA-B ΔC6L or mock-infected for 18 hours. The localization of C6 was analyzed by immunofluorescence, as described in *Materials and Methods*. Cells were staining with DAPI (blue, staining cellular nucleus), purified rabbit polyclonal anti-C6 (green) and anti-14K (red).

Western blot analyses using rabbit polyclonal antibodies raised against C6 (produced as described in *Material and Methods*) identified a 18.2 kDa protein in DF-1 cells infected with MVA-B, but not with MVA-B ΔC6L (Figure 2A). We then determined when C6 is expressed in DF-1 cells infected with WR and MVA (Figure 2B). C6 expression was detected at 3 hours post-infection, and increased with time for at least 22 hours. Treatment of DF-1 cells at the onset of infection with cytosine arabinoside (AraC), an inhibitor of viral DNA replication and therefore late protein expression, did not abrogate C6 expression, indicating that *C6L* is an early gene. The increased amount of early C6 protein at 22 h post-infection compared to what is produced when viral DNA replication is inhibited by AraC (Figure 2B), is probably due to enhanced stability of the protein after virus DNA synthesis and to the contribution of progeny virus.

The intracellular localization of C6 was examined by immunofluorescence in DF-1 cells infected with different VACV strains (Figure 2C). C6 was detected in the cytoplasm, presumably in viral factories, of DF-1 cells infected with WR, MVA and MVA-B, but not in MVA-B ΔC6L. The reduced fluorescence intensity of C6 (in green) indicates low levels of protein expression in comparison with the late protein A27 (in red).

### MVA-B ΔC6L up-regulates IFN-β expression in human macrophages and dendritic cells

**Figure 3:**
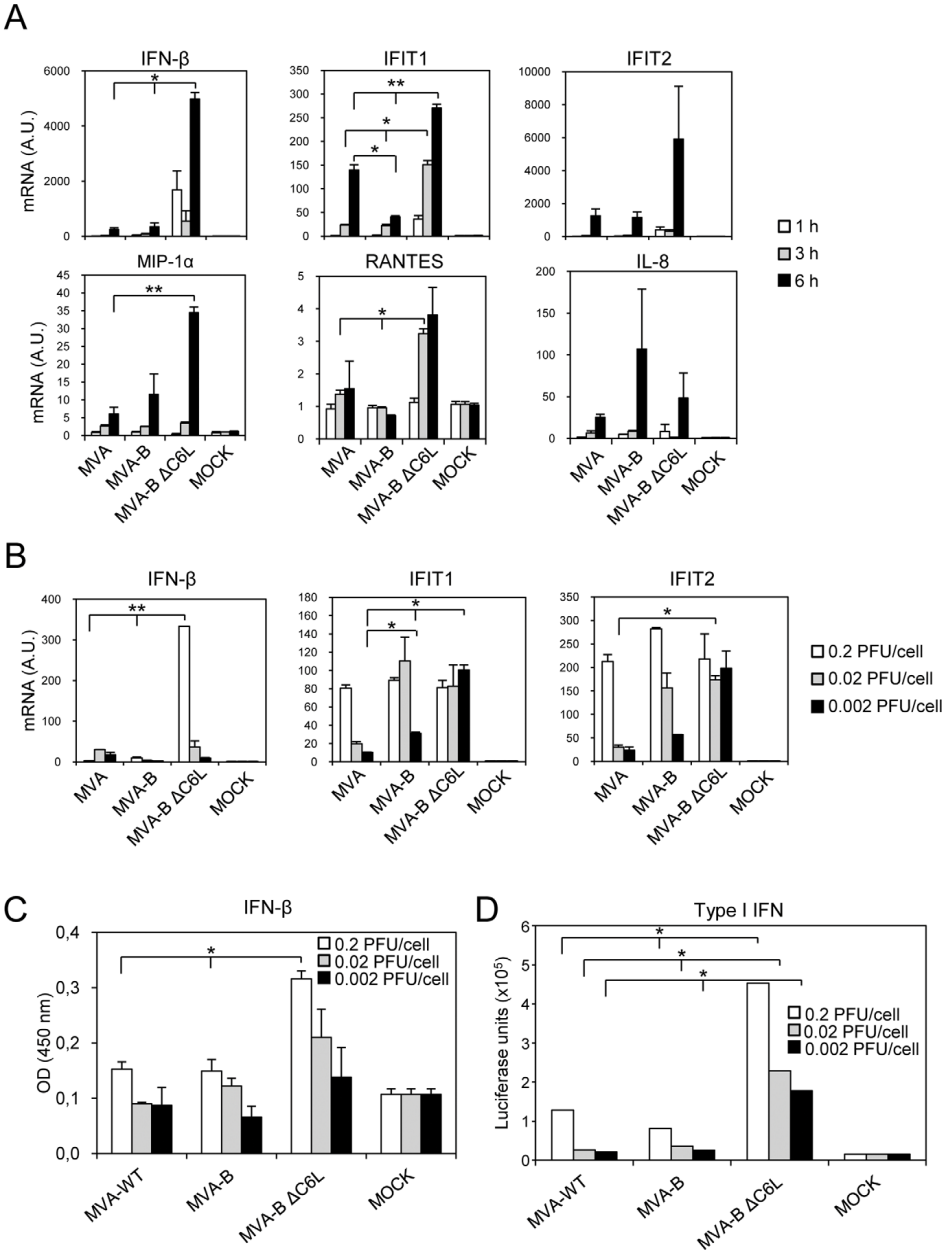
MVA-B ΔC6L induces the production of IFN-β and type I IFN inducible genes in macrophages and dendritic cells. Human THP-1 macrophages (A) and moDCs (B, C, D) were infected with MVA, MVA-B and MVA-B ΔC6L (5 PFU/cell in A, and 0.2, 0.02 and 0.002 PFU/cell in B, C and D). At different time post-infection (1 h, 3 h and 6 h in A, 6 h in B), RNA was extracted and the mRNA levels of IFN-β, type I IFN inducible genes (IFIT1 and IFIT2), chemokines and HPRT were analyzed by RT-PCR. Results were expressed as the ratio of gene to HPRT mRNA levels. A.U: arbitrary units. Data are means ± SD of duplicate samples. * *p*<0.05, ** *p*<0.005. (C, D) Human moDCs were infected with 0.2, 0.02 and 0.002 PFU/cell of MVA, MVA-B and MVA-B ΔC6L. Six hours later, cell-free supernatants were collected to quantify the concentration of IFN-β by ELISA (C) and the concentration of type I IFN using the HL116 reporter cell line (D). Results were expressed in absorbance values at 450 nm (C), and in luciferase units (D). Data are means ± SD of duplicates and are representative of two independent experiments. * *p*<0.05.

As a first step to determine whether C6 impairs the response of innate immune cells to MVA-B, we examined by real time PCR the expression of IFN-β, IFN-β-induced genes (IFIT1, IFIT2) and chemokines by human THP-1 macrophages infected for 1, 3 and 6 hours with MVA, MVA-B and MVA-B ΔC6L (Figure 3A). Compared to MVA and MVA-B, MVA-B ΔC6L (5 PFU/cell, Figure 3A and 1 PFU/cell, data not shown) markedly up-regulated IFN-β as well as IFIT1 and IFIT2 expression in THP-1 cells. MVA-B ΔC6L also increased the expression of MIP-1α and RANTES, but not that of IL-8 and IP-10 (Figure 3A and data not shown).

To confirm that C6 impaired IFN-β and IFN-β-dependent gene expression in innate immune cells, we infected human moDCs with increasing doses (0.002, 0.02 and 0.2 PFU/cell) of MVA, MVA-B and MVA-B ΔC6L and measured IFN-β, IFIT1 and IFIT2 mRNA levels 6 h post-infection (Figure 3B). We used low virus doses, since MVA induces apoptosis of human moDCs [26]. Similarly to the results obtained with human THP-1 cells, MVA-B ΔC6L strongly increased IFN-β expression compared to MVA and MVA-B in moDCs. Whereas the three viruses used at 0.2 PFU/ml similarly stimulated IFIT1 and IFIT2 mRNA expression in moDCs, MVA-B ΔC6L was a much more potent inducer than MVA and MVA-B at lower infective doses (0.002 PFU/ml, Figure 3B). Furthermore, MVA-B ΔC6L stimulated the release by moDCs of much higher levels of IFN-β (Figure 3C) and bioactive type I IFNs than MVA and MVA-B (Figure 3D).

Thus, deletion of *C6L* in the MVA-B genome promotes IFN-β production, suggesting that C6 interferes with the signalling pathway controlling IFN-β gene expression in innate immune cells.

### MVA-B ΔC6L enhances the magnitude and polyfunctionality of long-lived memory HIV-1-specific T-cell responses

Given the immunomodulatory properties of C6, we tested whether deletion of C6 in MVA-B ΔC6L could enhance its immunogenic properties by analyzing HIV-1-specific T-cell responses in BALB/c mice immunized with MVA-B or MVA-B ΔC6L using a DNA prime (100 µg of DNA-B, i.m.)/MVA boost (1×10^7^ PFU, i.p.) immunization protocol [17], [18], [22], [27], [28], [29]. Animals primed with sham DNA (DNA-φ) and boosted with non-recombinant MVA were used as controls. Considering that memory T-cell responses might be critical for protection against HIV-1 infection [30], [31], [32], [33], we assessed by IFN-γ ELISPOT and IFN-γ and IL-2 intracellular cytokine staining (ICS) the long-term immunogenicity profile (i.e. 53 days after the boost) elicited by DNA-B/MVA-B and DNA-B/MVA-B ΔC6L vaccination in splenocytes.

**Figure 4:**
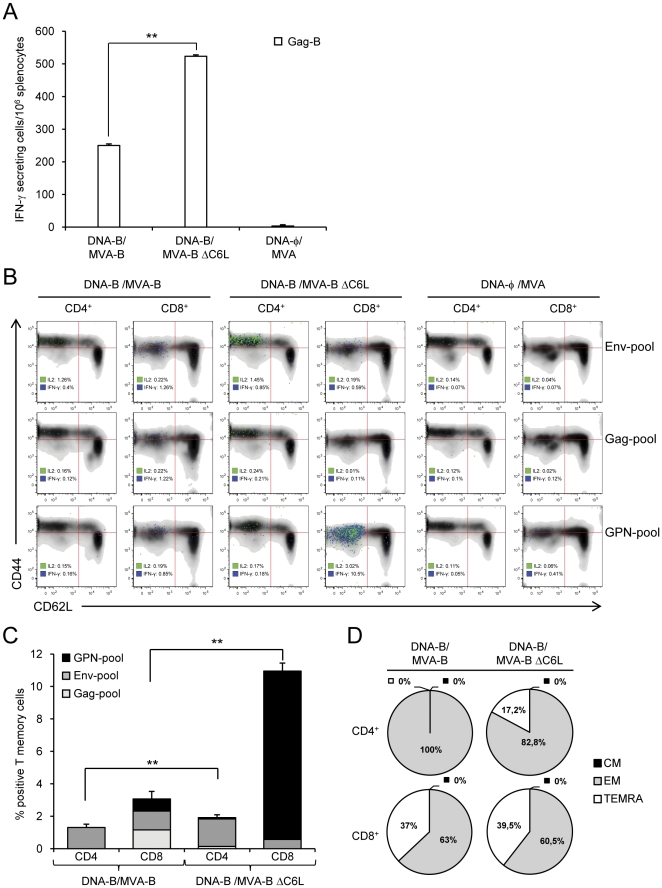
Immunization with MVA-B ΔC6L enhances the magnitude of HIV-1-specific CD4^+^ and CD8^+^ T-cell memory immune responses. Splenocytes were collected from mice (n = 4 per group) immunized with DNA-B/MVA-B, DNA-B/MVA-B ΔC6L or DNA-φ/MVA, 53 days after the last immunization. (A) Gag-B-specific IFN-γ secreting splenocytes were quantified by ELISPOT assay. Data are means ± SD of triplicate cultures. ** *p*<0.005. (B–D) Flow cytometry phenotypic analysis of Env, Gag and GPN HIV-1 specific CD4^+^ and CD8^+^ T-cells. CD44 and CD62L expression was used to identify central memory (CM: CD44^+^/CD62L^+^), effector memory (EM: CD44^+^/CD62L^−^) and effector memory terminally differentiated (TEMRA: CD44^−^/CD62L^−^) sub-populations. IFN-y and IL-2 production was analyzed by ICS. (B) A representative flow cytometry is shown. The T-cell memory sub-populations are depicted as density plots. Blue and green dots represent T-cells producing IFN-y and IL-2, respectively. (C) Percentage of splenic Env, Gag and GPN HIV-1-specific CD4^+^ and CD8^+^ memory T-cells. Frequencies were calculated by reporting the number of memory T-cells producing IFN-γ and/or IL-2 to the total number of CD4^+^ and CD8^+^ splenocytes. Values from unstimulated controls were subtracted in all cases. ** *p*<0.005. (D) Pie charts representing the proportion of CM, EM and TEMRA within the Env, Gag and GPN HIV-1-specific CD4^+^ and CD8^+^ memory T-cells. (A–D) Data are from one experiment representative of two experiments.

IFN-γ ELISPOT revealed that, compared to MVA-B, MVA-B ΔC6L enhanced 2.1-fold (*p*<0.005) the T-cell memory response against HIV-1 peptide Gag-B (an HIV-1 peptide representative of Gag antigen) (Figure 4A). Non-recombinant MVA, used as a control, did not induce HIV-1-specific memory responses.

The phenotype of the HIV-1-specific memory T cells elicited upon immunization with DNA-B/MVA-B and DNA-B/MVA-B ΔC6L was characterized by polychromatic flow cytometry using ICS. Splenic CD4^+^ and CD8^+^ T cells were co-stained for CD44 and CD62L surface markers to define the naïve (CD44^−^/CD62L^+^), central memory (CM: CD44^+^/CD62L^+^), effector memory (EM: CD44^+^/CD62L^−^) and effector memory terminally differentiated (TEMRA: CD44^−^/CD62L^−^) sub-populations. We also evaluated IFN-γ and IL-2 production after *in vitro* stimulation with different HIV-1 peptide pools (Env-pool, Gag-pool and GPN-pool) that covered the entire HIV-1 sequences present in the poxvirus vector (Figure 4B).

The overall HIV-1-specific immune response at 53 days post-boost was mainly mediated by CD8^+^ T cells (70%–85%) (Figure 4C) of EM and TEMRA phenotypes (Figure 4D), in both immunization groups. However, long-term post-boost immunization with DNA-B/MVA-B ΔC6L induced a higher magnitude of HIV-1-specific CD4^+^ and CD8^+^ T-cell memory responses producing IFN-γ and/or IL-2 than DNA-B/MVA-B [CD4^+^ T cells: 1.91% in DNA-B/MVA-B ΔC6L *vs.* 1.30% in DNA-B/MVA-B, (*p*<0.005); CD8^+^ T cells: 10.95% in DNA-B/MVA-B ΔC6L *vs.* 3.06% in DNA-B/MVA-B (*p*<0.005)] (Figure 4C). Both vectors induced a similar pattern of HIV-1-specific CD4^+^ T-cell memory responses (with preference towards Env) (Figure 4B and 4C). Interestingly, the pattern of CD8^+^ T-cell memory responses was different between the two vectors: DNA-B/MVA-B ΔC6L induced a higher percentage of GPN-specific CD8^+^ T-cell responses, while DNA-B/MVA-B induced preferentially Env- and Gag-specific CD8^+^ T-cell responses (Figure 4B and 4C). In both immunization groups, HIV-1-specific CD8^+^ T cells were mainly of the EM (60.5–63%) and TEMRA (37%–39.5%) phenotypes (Figure 4D). All HIV-1-specific CD4^+^ T cells were of the EM phenotype in the DNA-B/MVA-B group. Although most of HIV-1-specific CD4^+^ T cells (82.8%) were of the EM phenotype in the DNA-B/MVA-B ΔC6L group, a substantial proportion (17.2%) of cells expressed the TEMRA phenotype (Figure 4D). No CM T cells producing IFN-γ and/or IL-2 were detected in both immunization groups (Figure 4D).

**Figure 5:**
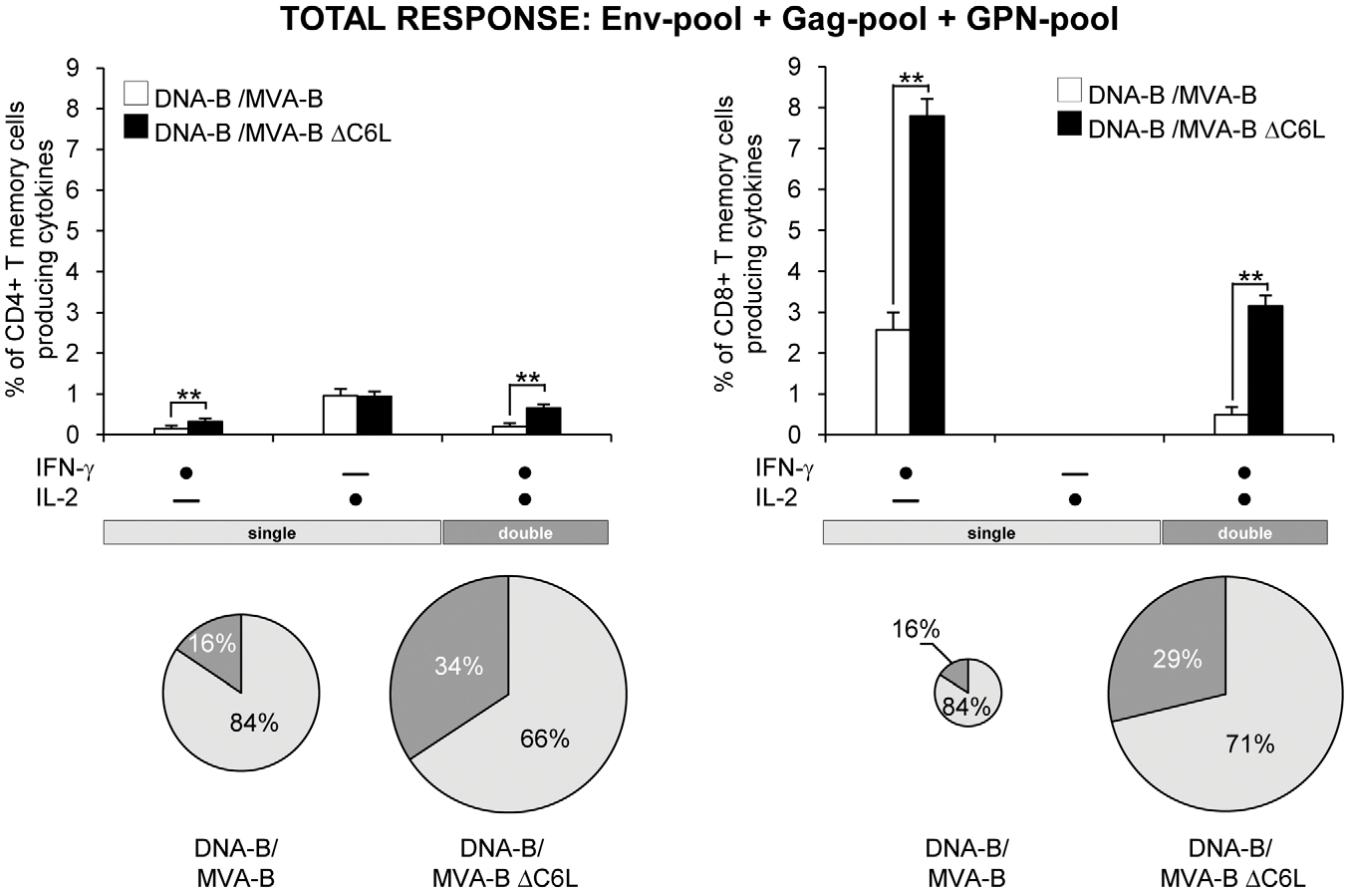
Immunization with MVA-B ΔC6L enhances the polyfunctionality of HIV-1-specific CD4^+^ and CD8^+^ T-cell memory immune responses. Splenocytes were collected from mice (n = 4 per group) immunized with DNA-B/MVA-B, DNA-B/MVA-B ΔC6L or DNA-φ/MVA, 53 days after the last immunization and analyzed by flow cytometry as described in Figure 4. The polyfunctionality of Env+Gag+GPN HIV-1-specific CD4^+^ (left part) and CD8^+^ (right part) memory T cells was defined based on IFN-γ and/or IL-2 production. All the possible combinations of the responses are shown on the X axis. The percentages of IFN-γ and/or IL-2 producing memory T-cells among total CD4^+^ and CD8^+^ T cells are shown on the Y axis. ** *p*<0.005. The pie charts summarize the data. Each slice corresponds to the proportion of CD4^+^ or CD8^+^ T cells producing single (IFN+ or IL-2+) or double (IFN+/IL-2+) responses within the total HIV-1-specific CD4^+^ or CD8^+^ T-cell memory populations. The size of the pie chart represents the magnitude of the specific HIV-1 memory immune response induced.

To have a detailed assessment of the quality of T-cell memory responses, we next evaluated the production of IFN-γ and/or IL-2 by HIV-1-specific CD4^+^ and CD8^+^ T-cell memory cells (Figure 5). DNA-B/MVA-B ΔC6L increased the polyfunctionality of HIV-1-specific CD4^+^ and CD8^+^ T memory cells consisting of cells producing both IFN-γ and IL-2 [CD4^+^ T cells: 34% in DNA-B/MVA-B ΔC6L *vs.* 16% in DNA-B/MVA-B, (*p*<0.005); CD8^+^ T cells: 29% in DNA-B/MVA-B ΔC6L *vs.* 16% in DNA-B/MVA-B, (*p*<0.005)] (Figure 5).

Altogether, these findings established that immunization with DNA-B/MVA-B ΔC6L significantly increased the magnitude and polyfunctionality of HIV-1-specific CD4^+^ and CD8^+^ T-cell memory responses, with most of the response mediated by EM and TEMRA T cells. HIV-1-specific CD4^+^ T-cell memory responses were preferentially Env-specific following DNA-B/MVA-B and DNA-B/MVA-B ΔC6L vaccination. Yet, DNA-B/MVA-B ΔC6L induced an immunodominance towards CD8^+^ GPN-specific T-cell memory responses, while DNA-B/MVA-B induced preferentially CD8^+^ Env- and Gag-specific T-cell memory responses.

### MVA-B ΔC6L enhances the levels of antibodies against HIV-1 gp120

**Figure 6:**
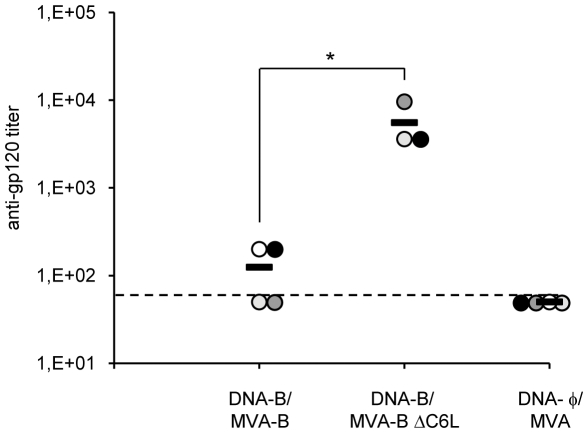
Immunization with MVA-B ΔC6L enhances the humoral immune responses elicited against HIV-1 gp160 protein. Serum was collected from individual mouse immunized with DNA-B/MVA-B, DNA-B/MVA-B ΔC6L or DNA-φ/MVA (n = 4 in DNA-B/MVA-B and DNA-φ/MVA; n = 3 in DNA-B/MVA-B ΔC6L), 53 days after the last immunization. Anti-gp120 antibody titers were determined by ELISA as described in *Materials and Methods*. Titers represent the last dilution of the serum that signals 3-fold higher than signals obtained with the serum of naïve mice. The dotted line represents the limit of detection of the ELISA. The horizontal bar represents the mean value. Each dot represents one mouse. * *p*<0.05.

Since cells infected with MVA-B release monomeric gp120 [18], we evaluated whether DNA-B/MVA-B and DNA-B/MVA-B ΔC6L immunization stimulated the production of antibodies against HIV-1 Env. Anti-gp120 antibodies in serum from individual mouse collected 53 days post-boost were quantified by ELISA, measuring the levels of specific antibodies reactive against gp160 protein from the HIV-1 clone LAV (clade B). Compared to DNA-B/MVA-B, DNA-B/MVA-B ΔC6L immunization increased 44-fold the levels of antibodies reactive against gp160 protein (Figure 6). Therefore, MVA-B ΔC6L increases the humoral immune responses against HIV-1 Env.

## Discussion

The MVA vector, despite of its attenuated phenotype, still contains genes that encode proteins that can interfere with host immune responses to viral infection [34], and it is described that deletion of immunomodulatory proteins in orthopoxviruses can enhance immune responses ([17], [34], [35], [36], [37], [38], [39]). The function of some of these genes, like the VACV gene *C6L*, is unknown. We report here the immunomodulatory role of *C6L*, showing the effects of the C6 protein on virus replication, innate immune sensing and immunogenicity *in vivo*.

MVA-B, the attenuated VACV vector MVA expressing the clade B HIV-1 antigens Env, as monomeric gp120, and Gag, Pol and Nef, as a polyprotein of about 160 kDa is considered a vaccine candidate against HIV/AIDS [18] based on preclinical studies in different animal models [17], [18], [20], [22] and on gene signatures triggered in human DCs infected with MVA-B, where the expression of HIV-1 proteins induced the expression of immunomodulatory molecules such as cytokines, cytokine receptors, chemokines, chemokine receptors and molecules involved in antigen uptake and processing [24]. Moreover, human DCs exposed to MVA-B induced highly functional HIV-1-specific CD8^+^ T-cell responses in HIV-1 infected individuals [25]. Thus, due to the good immunogenicity behavior of MVA-B, a prophylactic phase I clinical trial was initiated in Spain.

To improve the immunogenicity elicited by MVA-B and to investigate the possible immunomodulatory role of *C6L* we have removed from the MVA-B viral genome the *C6L* gene, generating the deletion mutant termed MVA-B ΔC6L. First, we showed in cultured cells that MVA-B ΔC6L does not express the C6 protein, but efficiently produced the four HIV-1 antigens (Env, Gag, Pol and Nef) in a stable manner and at the same level as MVA-B during the course of virus infection. Also, MVA-B ΔC6L replicates similarly to MVA-B in cultured cells, indicating that deletion of *C6L* has no effect on virus propagation. Therefore, *C6L* is not essential for viral replication in cell culture. Moreover, similar to MVA-B, MVA-B ΔC6L maintains an attenuated phenotype and does not replicate in mammalian cells. Western blot analyses demonstrated that C6 is expressed early in cells infected with the VACV strains WR and MVA. This early expression profile is consistent with genome-wide transcriptome analyses that detected C6 mRNA 30 minutes post-infection [2]. Most VACV immunomodulatory proteins are expressed early during infection, and the early expression pattern of C6 suggests that it is involved in immune evasion as we confirmed in experiments using human macrophages and DCs. In addition, C6 localizes to the cytoplasm of infected cells, opening the possibility that C6 modulates, directly or indirectly, intracellular signalling pathways controlling immune responses.

Yeast two-hybrid and pull-down assays revealed that VACV C6 protein binds to three host human cell proteins [14]. However, none of these proteins seems to be directly related with the host immune response. One of the C6 binding partners is programmed cell death 6 interacting protein (PDCD6IP/ALIX), which has been involved in the regulation of apoptosis, cytokinesis and HIV-1 budding. VACV C6 also interacts with keratin 4 (KRT4), present in intermediate filaments, and which also binds IMV surface protein A27. C6 protein has also been detected in a low proportion in intracellular mature virions [13], similar to other proteins of the poxvirus family Pox_A46 (A46). One possible reason for presence of C6 in the virion could be that C6 is necessary for viral cycle early after virus entry or that C6 have a function in IMV-cell attachment, fusion, and/or microtubule transport through their interaction with KRT4. Finally, C6 also binds to troponin I, skeletal, fast (TNNI2), a co-activator of estrogen receptor-related receptor α (ERRα), suggesting that C6 could have a role in ERRα-mediated transcriptional activity. Additional experiments will be required to decipher the relationship between the C6 interaction with binding partners and C6 immunomodulatory function.

A bioinformatic analysis indicated that *C6L* has sequence similarities with the poxvirus family Pox_A46, a poxvirus Bcl-2-like gene family, which includes *A46R*, *A52R*, *K7R* and *B15R* (named *B14R* in VACV strain WR) [3]. A46 [4], [12], A52 [4], [7], [8], K7 [9], [10], [11] and B15 [5], [6] are intracellular proteins expressed by VACV that inhibit TLR signalling at different levels. A46 contains a Toll/IL-1 receptor (TIR) domain and targets several TIR adaptor proteins (MyD88, TIRAP, TRIF and TRAM) [4], [12], blocking MAP kinase activation and TRIF-mediated IRF3 activation. A52 and K7 targets IRAK2 and TRAF6 inhibiting TLR-dependent NF-κB activation [11]. K7 also interacts with DDX3, which is part of the complex that activates transcription factor IRF3, thus inhibiting IRF3 mediated IFN-β gene transcription [9], [10]. B15 inhibits IKKβ phosphorylation thereby impairing NF-κB activation [5], [6]. Considering the sequence similarities of C6 with A46, A52, K7 and B15, we speculate that C6 may interfere with host immune responses through inhibition of TLR signalling pathways.

To examine the immunomodulatory role of *C6L*, we characterized the profile of the innate immune sensing induced by MVA-B ΔC6L, MVA-B and MVA in human macrophages and moDCs. MVA-B ΔC6L significantly up-regulated IFN-β and IFN-α/β-inducible genes (IFIT1 and IFIT2) mRNA levels and increased IFN-β secretion, suggesting that C6 blocks some component of the IFN-β signalling pathway. The effect was mainly observed 6 hours post-infection and at low virus doses, probably because long-term viral exposure and high viral doses induce extensive apoptosis [26]. Previous experiments revealed in macrophages infected with MVA a critical role for TLR2-TLR6-MyD88 in the production of IFN-β-independent chemokines and of MDA-5-IPS-1 in the production of IFN-β-dependent chemokines [40]. Phosphorylation of IRF3, IRF7 and STAT-1, which are essential for the transcription of the IFN-β gene (IRF3) and critical targets of the IFN-β signalling pathway required for the transcriptional activation of IFN-β-dependent genes (IRF7 and STAT-1), were detected in THP-1 cells infected with MVA and associated with the induction of IFN-β [40]. Whether C6 blocks directly or indirectly IRF3, IRF7 or STAT-1 expression or activation by phosphorylation is under investigation.

Since our goal is to develop modified MVA-B with enhanced immunogenicity to HIV-1 antigens, we carried out a detail characterization of the HIV-1-specific memory immune responses induced in mice using DNA prime/MVA boost approach, and compared parental MVA-B with the deletion mutant MVA-B ΔC6L. We used ICS, which allows a more extensive characterization of T-cell effector functions at the single-cell level [41]. Our findings revealed that at 53 days post-boost, DNA-B/MVA-B ΔC6L triggered higher magnitude and polyfunctionality of total HIV-1-specific CD4^+^ and CD8^+^ T-cell memory immune responses (specific for Env, Gag and GPN) than DNA-B/MVA-B. The vaccine-induced T-cell memory responses were predominantly mediated by CD8^+^ T cells in both immunization groups, with most of the response mediated by CD8^+^ EM and TEMRA T cells, which have been described to have a powerful and direct antiviral capacity [30], [31], [32], [33] and have been associated with HIV-1 viral control in early and chronic infection [42], [43], [44]. HIV-1-specific CD4^+^ T-cell memory responses were preferentially Env-specific in both immunization groups, similar to what it is obtained with other MVA HIV-1 vaccines as MVA-CMDR [45] or other NYVAC HIV-1 vaccines [21], [46], [47]. Furthermore, immunization with DNA-B/MVA-B ΔC6L induced preferentially GPN-specific CD8^+^ T-cell memory responses compared with DNA-B/MVA-B. The shift towards GPN-response triggered by MVA-B ΔC6L might result from the activation of the intrinsic pathway of antigen presentation by the Gag-Pol-Nef intracellular polyprotein due to the increased IFN-β production promoted by the deletion of *C6L*. However, it is not clear yet whether the enhanced CD8^+^ T-cell response to GPN is due to a greater breadth of response or could reflect an enhanced response to single epitopes. The functional relevance of Env-specific CD4^+^ T-cell responses or GPN-specific CD8^+^ T-cell responses in setting of vaccination for prevention of HIV-1 infection needs to be further explored. Further experiments need to be done in other animal models such as non-human primates to determine the possible benefits of the response elicited by MVA-B ΔC6L. Interestingly, enhanced Gag response has been associated with better control of virus in macaques infected with SIV and in HIV-1-infected individuals [48], [49].

How significant are our *in vivo* findings with regard to immune requirements for HIV-1 protection? While definition of correlates of protection to HIV-1 remains to be firmly established, there are a number of markers that can be used as potential indicators for an effective HIV-1 vaccine, such as: 1) activation of HIV-1-specific CD4^+^ and CD8^+^ T cells; 2) triggering polyfunctional responses; 3) enhanced magnitude and breath of the immune response; 4) induction of long-term memory cells of effector phenotype; 5) production of neutralizing antibodies with broad specificities. Several features of the T-cell response to HIV-1 are correlated with control of viral replication [50], [51], and a correlation of the CD8^+^ T-cell response with a lowering of peak viremia in acute HIV-1 infection has been described [52], [53]. Also, in non-human primates there is a good correlation between vaccine-induced HIV-1-specific cellular immunogenicity and protection after a challenge with a pathogenic SHIV [22], [27], [28], where CD8^+^ T cells play an important role in immunity to HIV-1 [54]. Several studies have demonstrated that T-cell polyfunctionality is associated with protective antiviral immunity [[50], [51], [55], [56], [57], for a review [33]]. In HIV-1-infected patients that are nonprogressors, HIV-1-specific CD8^+^ T cells were polyfunctional [50]. Furthermore, the generation of memory CD8^+^ T cells of EM and TEMRA phenotypes have been associated with the control of HIV-1 infection in patients [42], [43], [44] and in non-human primates [58]. Moreover, it was described recently that effector memory T-cell responses elicited after vaccination in non-human primates could control highly pathogenic SIVmac239 infection early after mucosal challenge, showing the important role of memory T-cell responses [59]. These observations suggest that polyfunctional CD8^+^ T cells and effector memory T cells are important components of a protective immune response [33]. Importantly, both MVA-B and MVA-B ΔC6L triggered immune responses that fulfill several of the characteristics of a promising candidate HIV-1 vaccine. Indeed immunization with DNA-B/MVA-B ΔC6L induced activation of HIV-1-specific CD4^+^ and CD8^+^ T cells, enhanced magnitude and polyfunctionality of the immune response, triggered long-term memory T cells of effector phenotype, and increased the levels of antibodies directed against Env.

However, the immunological parameters required for protection against HIV-1 infection in humans remain unknown. The phase III Thai clinical trial reported a modest protection of about 31% against HIV-1 infection in vaccinees with the combination of recombinant vaccines canarypox and gp120, in spite of poor neutralizing antibodies and of reduced T-cell responses against HIV-1 [60]. The phase III Thai trial has pointed out that further developments of poxvirus vectors is needed. Among MVA vectors, several phase I clinical studies for HIV/AIDS have been performed with DNA prime/MVA boost protocols or with MVA administered alone [61], [62], [63], [64], [65] and revealed promising findings. These studies proved the safety and immunogenicity of the MVA vectors and reported an important proportion of responders with multigenic responses that persisted up to one year post-vaccination. In light of our results, one might postulate that MVA-B with deletion of the *C6L* gene could improve the immunogenicity of the vaccines by enhancing the magnitude, polyfunctional and memory responses T cell responses.

In conclusion, we show that deletion of *C6L* on MVA-B up-regulates IFN-β expression in human macrophages and DCs, and improves MVA-B immunogenicity *in vivo*, increasing the magnitude, polyfunctionality and memory T-cell responses against HIV-1 and the generation of Env-specific antibodies. Thus, VACV C6 protein interferes with host immune responses by at least in part, blocking some component(s) of the IFN-β signalling pathway. Understanding the mechanism of action of C6 will provide new insights in virus-host cell interactions and viral immunomodulation. Further work should be devoted to explore the relevance of our findings in a non-human primate model, as MVA-B ΔC6L represents a promising vector for developing HIV-1 vaccines.

## Materials and Methods

### Ethics Statement

The animal studies were approved by the Ethical Committee of Animal Experimentation (CEEA-CNB) of Centro Nacional de Biotecnologia (CNB-CSIC, Madrid, Spain) in accordance with national and international guidelines and with the Royal Decree (RD 1201/2005). Permit numbers: 152/07 and 080030.

Studies with peripheral blood mononuclear cells (PBMCs) from healthy blood donors recruited by the Blood Center of Lausanne (Switzerland) were approved by the ethics commission for clinical research from the Faculty of Biology and Medicine of Lausanne. Written informed consent was obtained from donors. All information were kept confidential by the Blood Center.

### Cells and viruses

Primary chicken embryo fibroblast cells (CEF) [18] and DF-1 cells (a spontaneously immortalized chicken embryo fibroblast cell line. ATCC, Manassas, VA) were grown in Dulbecco's modified Eagle's medium (DMEM) supplemented with 10% fetal calf serum (FCS). The human monocytic THP-1 cell line (ATCC, Manassas, VA) was cultured in complete RPMI 1640 medium containing 2 mM L-glutamine, 50 µM 2-mercaptoethanol, 100 IU/ml penicillin, 100 µg/ml streptomycin (all from Invitrogen, San Diego, CA) and 10% heat-inactivated FCS (Sigma-Aldrich, St. Louis, MO), as previously described [40]. THP-1 cells were differentiated into macrophages by treatment with 0.5 mM phorbol 12-myristate 13-acetate (PMA, Sigma-Aldrich) for 24 h before usage. Adult peripheral blood mononuclear cells (PBMCs) from healthy donors (recruited by the Blood Center, Lausanne, Switzerland) were seeded in 6-well tissue culture plates (3×10^6^ cells/well) in complete RPMI medium supplemented with 10% heat-inactivated FCS and incubated at 37°C for 3 h. Non-adherent cells were removed and medium replaced by fresh complete RPMI medium containing 50 ng/ml granulocyte-macrophage colony-stimulating factor (GM-CSF) and 20 ng/ml IL-4 (R&D Systems, Minneapolis, MN). moDCs were collected after 7 days of incubation. Cell cultures were performed at 37°C (CEF, THP-1 cells and moDCs) or 39°C (DF-1) in a humidified incubator containing 5% CO_2_.

The poxvirus strains used in this work included: Western Reserve (WR), modified vaccinia virus Ankara (MVA) and the recombinant MVA-B expressing the HIV-1_BX08_ gp120 and HIV-1_IIIB_ Gag-Pol-Nef proteins [18]. Viruses were grown in CEF cells, purified through two 36% (w/v) sucrose cushions, and titrated by plaque immunostaining assay [66]. Cell lines were infected with viruses as previously described [18], [40].

### Construction of plasmid transfer vector pGem-RG-C6L wm

The plasmid transfer vector pGem-RG-C6L wm was used for the construction of the recombinant virus MVA-B ΔC6L, with *C6L* (*C6L* in Copenhagen strain of VACV is equivalent to *MVA 019L* in MVA) gene deleted (for simplicity, we used throughout the work the ORF nomenclature of Copenhagen strain to refer the MVA genes). pGem-RG-C6L wm was obtained by sequential cloning of five DNA fragments containing dsRed2 and rsGFP genes and *C6L* recombination flanking sequences into the plasmid pGem-7Zf(−) (Promega). The construction of the plasmid pGem-Red-GFP wm (4540 bp), containing dsRed2 and rsGFP genes under the control of the synthetic early/late (E/L) promoter was previously described [17]. MVA-B genome was used as the template to amplify the right flank of *C6L* gene (391 bp) with oligonucleotides RFC6L-AatII-F (5′-CTCGTCGACGTCCGACCAATCTGGGC-3′) (AatII site underlined) and RFC6L-XbaI-R (5′-TTCCTATCTAGATTTCTCTGTTTAAA-3′) (XbaI site underlined). This right flank was digested with AatII and XbaI and cloned into plasmid pGem-Red-GFP wm previously digested with the same restriction enzymes to generate pGem-RG-RFsC6L wm (4898 bp). The repeated right flank of *C6L* gene (391 bp) was amplified by PCR from MVA-B genome with oligonucleotides RF′C6L-XmaI-F (5′-CTCGTCCCCGGGCGACCAATCTGGGC-3′) (XmaI site underlined) and RF′C6L-ClaI-R (5′-TTCCTAATCGATTTTCTCTGTTTAAA-3′) (ClaI site underlined), digested with XmaI and ClaI and inserted into the XmaI/ClaI-digested pGem-RG-RFsC6L wm to generate pGem-RG-RFdC6L wm (5259 bp). The left flank of *C6L* gene (413 bp) was amplified by PCR from MVA-B genome with oligonucleotides LFC6L-ClaI-F (5′-ATACGCATCGATGATAAACTTAATGA-3′) (ClaI site underlined) and LFC6L-BamHI-R (5′-GTTGTTGGATCCATTGGTAGATGACG-3′) (BamHI site underlined), digested with ClaI and BamHI and inserted into the ClaI/Bam HI-digested pGem-RG-RFdC6L wm. The resulting plasmid pGem-RG-C6L wm (5642 bp) was confirmed by DNA sequence analysis and directs the deletion of *C6L* gene from MVA-B genome.

### Construction of MVA-B ΔC6L deletion mutant

MVA-B ΔC6L deletion mutant was constructed by screening for transient Red2/GFP co-expression using dsRed2 and rsGFP genes as the transiently selectable markers, as previously described [17]. Briefly, 3×10^6^ DF-1 cells were infected with MVA-B at a multiplicity of 0.05 PFU/cell and then transfected 1 h later with 6 µg of DNA from plasmid pGem-RG-C6L wm using Lipofectamine (Invitrogen) according to the manufacturer's recommendations. After 72 hours, the cells were harvested, lysed by freeze-thaw cycling and sonicated. Following 6 consecutive rounds of plaque purification in DF-1 cells, MVA-B ΔC6L was obtained and the deletion of *C6L* gene was confirmed by PCR amplifying the *C6L* locus. MVA-B ΔC6L was grown in CEF cells, purified by centrifugation through two 36% (w/v) sucrose cushions in 10 mM Tris-HCl pH 9, and titrated in DF-1 cells by plaque immunostaining assay, using rabbit polyclonal antibody against VACV strain WR (Centro Nacional de Biotecnología; 1∶1000) followed by anti-rabbit-HRP (Sigma; 1∶1000), as previously described [66]. MVA-B ΔC6L deletion mutant was free of contamination with mycoplasma or bacteria.

### PCR analysis of MVA-B ΔC6L deletion mutant

To test the purity of MVA-B ΔC6L deletion mutant, viral DNA was extracted from DF-1 cells mock-infected or infected at 2 PFU/cell with MVA, MVA-B or MVA-B ΔC6L. Primers RFC6L-AatII-F and LFC6L-BamHI-R (described above) spanning *C6L* flanking regions were used for PCR analysis of *C6L* locus. The amplification protocol was previously described [17]. PCR products were resolved in 1% agarose gel and visualized by ethidium bromide staining. The *C6L* deletion was also confirmed by DNA sequence analysis.

### Expression of HIV-1_BX08_ gp120 and HIV-1_IIIB_ Gag-Pol-Nef proteins by MVA-B ΔC6L deletion mutant

To test the correct expression of HIV-1 proteins HIV-1_BX08_ gp120 and HIV-1_IIIB_ Gag-Pol-Nef (GPN), monolayers of DF-1 cells were mock-infected or infected at 2 PFU/cell with MVA, MVA-B or MVA-B ΔC6L. After 24 hours, cells were lysed in Laemmli buffer, cells extracts were fractionated in 12% SDS-PAGE and analyzed by Western blot using rabbit polyclonal anti-gp120 antibody against IIIB (Centro Nacional de Biotecnología; 1∶3000) or polyclonal anti-gag p24 serum (ARP 432, NIBSC, Centralised Facility for AIDS reagent, UK; 1∶3000) followed by anti-rabbit-HRP (Sigma; 1∶5000) to evaluate the expression of gp120 and GPN proteins, respectively.

### Analysis of virus growth

To determine virus-growth profiles, monolayers of DF-1 cells grown in 12-well tissue culture plates were infected in duplicate at 0.01 PFU/cell with MVA-B or MVA-B ΔC6L. Following virus adsorption for 60 min at 37°C, the inoculum was removed. The infected cells were washed once with DMEM without serum and incubated with fresh DMEM containing 2% FCS at 37°C in a 5% CO_2_ atmosphere. At different times post-infection (0, 24, 48 and 72 hours), cells were collected, freeze-thawed three times and briefly sonicated. The intracellular viruses were titrated by immunostaining as described above.

### Expression of C6 protein in *E. coli* and production of anti-C6 polyclonal antibodies

The C6 ORF (*MVA 019L*, 157 aa, 18.2 kDa) was amplified by PCR using primers C6L-NheI-F (5′-AGGCTAGCGTTTAGGAAAAAAAAATATC-3′) (NheI site underlined) and C6L-BamHI-R (5′-AAGGATCCCATGAATGCGTATAATA-3′) (BamHI site underlined), and VACV MVA DNA as template. The product (488 bp) was digested with NheI and BamHI and cloned into plasmid pET-27b(+) (Novagen). The ligation product was used to transform BL21 *E.coli*, and the plasmid of a kanamycin-resistant positive colony was sequenced to confirm that it contained the *C6L* sequence. The plasmid generated was termed pET-27b-C6L (5837 bp). Plasmid pET-27b(+) provided a tract of 6 histidines at the carboxyl terminus of the C6 protein, generating a recombinant C6 protein of about 24 kDa. Kanamycin-resistant colonies were grown in Luria broth medium until an OD of 0.5 at 595 nm. Isopropyl 1-thio-β-D-galactopyranoside was added (0.5 mM) and the culture grown for 4 additional hours. Cells were pelleted by centrifugation. For lysis, cells were suspended in 50 mM Tris–HCl, pH 7.5, 0.3 M NaCl, 8 M Urea, and incubated with 1 mg/ml lysozyme for 30 min in the presence of 1 mM phenylmethylsulfonyl fluoride. The suspension was freezed-thawed twice. Cellular debris was removed by centrifugation, and the supernatant was incubated with Probound resin (Invitrogen). Elution was carried out with different concentrations of imidazol (100 to 500 mM) in 50 mM Tris–HCl, pH 7.5, 0.3 M NaCl. Eluted fractions were pooled and loaded on desalting columns following the manufacturer's protocol (GE-Healthcare, Freiburg, Germany), and fractions were collected. Protein was quantified using the Bradford assay, fractionated by 12% SDS-PAGE and analyzed by Western blot using anti-His tag antibody (1∶5000) to detect the presence of VACV C6 protein. Fractions containing the C6 protein (with an estimated purity of 90%) were stored in aliquots at −20°C. The C6 protein (1150 µg) was injected into New Zealand White rabbits to produce anti-C6 serum and rabbit polyclonal antibody against C6 (Biomedal Laboratories, Sevilla, Spain).

### Early expression of C6 protein in cells infected by WR and MVA

DF-1 cells were infected with WR and MVA at 5 PFU/cell in the presence or absence of 40 µg/ml of cytosine arabinoside (AraC; an inhibitor of viral DNA replication and therefore late protein expression). At different times post-infection (30 min, 3 h, 6 h and 22 h) cells were lysed in Laemmli buffer, cells extracts fractionated by 12% SDS-PAGE and analyzed by Western blot using rabbit polyclonal sera anti-C6 (Biomedal; 1∶100), followed by anti-rabbit-HRP (Sigma; 1∶5000). Expression of C6 protein was analyzed also 22 h post-infection in DF-1 cells infected at 5 PFU/cell with MVA-B and MVA-B ΔC6L.

### Immunofluorescence

DF-1 cells were grown on glass coverslips (borosilicate glass; BDH) in 12-well plates and infected at 0.5 PFU/cell with WR, MVA, MVA-B or MVA-B ΔC6L. At 18 h post-infection cells were washed three times in ice-cold PBS, fixed in 4% paraformaldehyde, permeabilized with 0.1% Triton X-100 (Sigma), and blocked with 10% FCS. Cells were incubated with antibodies to the VACV C6 viral protein (rabbit polyclonal anti-C6, 1∶500), and A27L viral protein (mouse monoclonal antibody C3anti-14K, 1∶400), together with the DNA-staining reagent DAPI (1∶200) at RT for 1 h. Samples were incubated with the secondary antibodies (Alexa-488 Goat anti-rabbit IgG and Alexa-546 Goat anti-mouse IgG; 1∶500) at RT for 1 h, washed and mounted in ProLong™ Antifade Kit medium. Images were obtained with a Bio-Rad Radiance 2100 confocal laser microscope.

### RNA analysis by quantitative real-time polymerase chain reaction

Total RNA was isolated from THP-1 cells and moDCs infected with MVA, MVA-B and MVA-B ΔC6L, using the RNeasy kit (Qiagen, Hombrechtikon, Switzerland). Reverse transcription of 100 ng to 500 ng of RNA was performed using the ImProm II RT System kit (Promega). Quantitative PCR was performed with a 7500 Fast Real-Time PCR System (Applied Biosystems, Rotkreuz, Switzerland) using the Power SYBR Green PCR Master Mix (Applied Biosystems), as previously described [40]. Expression levels of *IFN-β, IFIT1, IFIT2, RANTES, MIP-1α, IL-8, IP-10 and HPRT* genes were analyzed by real-time PCR using specific oligonucleotides (sequence be provided upon request). Gene specific expression was expressed relative to the expression of *HPRT* in arbitrary units (A.U.). All samples were tested in duplicates.

### IFN-β and type I IFN measurement

IFN-β concentrations in cell-culture supernatants were measured by ELISA (PBL Biomedical Laboratories, Picataway, NJ). Type I IFNs were quantified as previously described using the reporter cell line HL116 which stably expresses the *luciferase* gene under the control of the IFN-α/β-inducible 6–16 promoter [67], [68], [69]. HL116 cells were grown in DMEM supplemented with 10% FCS and 50 mM hypoxanthine-aminopterin-thymidine. HL116 cells (10^5^ cells in a 96-well plate) were incubated for 6 h with cell culture supernatants or recombinant human IFN-β (Cell Sciences, Canton, MA) as a standard. Luciferase activity in HL116 lysates was quantified using the Luciferase assay reagent (Promega) and a luminometer (EG&E Berthold, Germany).

### Peptides

HIV-1 peptide pools were provided by the EuroVacc Foundation. They spanned the entire Env, Gag, Pol and Nef regions from clade B HIV-1 as consecutive 15-mers overlapped by 11 amino acids. The HIV-1_BX08_ gp120 protein was spanned by the Env-1 and Env-2 pools. The HIV-1_IIIB_ Gag-Pol-Nef fusion protein was spanned by the following pools: Gag-1, Gag-2, GPN-1, GPN-2, GPN-3 and GPN-4. The size and number of peptides included in each pool was previously described [17]. For immunological analysis we grouped the peptides in three main pools: Env, Gag and GPN. The Env-pool comprises Env-1+Env-2; Gag-pool comprises Gag-1+Gag-2; and GPN-pool comprises GPN-1+GPN-2+GPN-3+GPN-4. HIV-1 peptide Gag-B (AMQMLKETI), from clade B, was produced at Centro Nacional de Biotecnología.

### Mice immunization schedule

BALB/c mice (6–8 weeks old) were purchased from Harlan. A DNA prime/MVA boost immunization protocol was performed as previously described [17], [18]. Groups of animals (n = 4) received 100 µg of DNA-B (50 µg of pCMV-_BX08_gp120+50 µg of pCDNA-_IIIB_GPN) by intramuscular route (i.m.) and two weeks later received an intraperitoneal (i.p.) inoculation of 1×10^7^ PFU of the corresponding recombinant vaccinia viruses (MVA-B or MVA-B ΔC6L) in 200 µl of PBS. Mice immunized with sham DNA (DNA-φ) followed by MVA booster were used as control group. At 53 days after the last immunization, mice were sacrificed and spleens processed for IFN-γ ELISPOT and intracellular cytokine staining (ICS) assays. Two independent experiments have been performed.

### IFN-γ ELISPOT assay

IFN-γ ELISPOT assay was performed as previously described [17], [70].

### Intracellular Cytokine Staining assay (ICS)

The phenotypes of responding T memory cells were analyzed by ICS and fluorescence-activated cell sorting analysis as described elsewhere [22]. After an overnight rest, 5×10^6^ splenocytes (depleted of red blood cells) were resuspended in RPMI 1640 supplemented with 10% FCS and containing 1 µl/ml Golgiplug (BD Biosciences) to inhibit cytokine secretion. Cells were seeded on M96 plates and stimulated with Env-, Gag- or GPN-pools of peptides (5 µg/ml). After 6 h of stimulation, cells were washed, stained with anti-CD4-Alexa 700, -CD8-FITC, -CD44-PECy5 and -CD62L-PE antibodies, fixed, permeabilized using the BD Cytofix/Cytoperm™ Kit (Becton Dickinson) and stained with anti-FN-γ-PECY-7 and anti-IL-2-Alexa-647 antibodies (all antibodies were from BD Biosciences). Dead cells were excluded using the violet LIVE/DEAD stain kit (Invitrogen). Acquisition and analyses were performed using a LSRII flow cytometer (Becton Dickinson) and FlowJo version 8.5.3 (Tree Star, Ashland, OR). The production of IFN-γ and IL-2 was analyzed in each of the T-cell memory populations: central memory (CM: CD44^+^/CD62L^+^), effector memory (EM: CD44^+^/CD62L^−^) and effector memory terminally differentiated (TEMRA: CD44^−^/CD62L^−^) sub-populations.

### Antibody measurements by ELISA

Antibodies anti-HIV-1 gp160LAV envelope protein were measured by ELISA as previously described [17], [18].

### Statistical procedures

The statistical analysis of ELISPOT and ICS assays was realized as previously described [17], [71]. We have developed a novel approach that corrects measurements for the medium response (RPMI), calculating confidence intervals and p-values. Only antigen responses values significantly larger than the corresponding RPMI are represented.

## Supporting Information

Figure S1 **Virus growth of MVA-B and MVA-B ΔC6L in HeLa cells.** Monolayers of HeLa cells were infected at 0.01 PFU/cell with WR, MVA-B or MVA-B ΔC6L for 0, 24, 48, and 72 h. For comparative purposes, we used the replication-competent WR strain. Cells were collected by centrifugation and infectious viruses associated with the cells (intracellular) and released to the medium (extracellular) during the course of the infection were measured by a plaque immunostaining assay with anti-WR antibodies. Data are from one experiment representative of two experiments.
(TIF)
