# Supplementary figures and images for: A Candidate HIV/AIDS Vaccine (MVA-B) Lacking Vaccinia Virus Gene C6L Enhances Memory HIV-1-Specific T-Cell Responses
Source: PLoS One. 2011 Aug 31;6(8):e24244. doi: 10.1371/journal.pone.0024244 (PMC3164197; doi:10.1371/journal.pone.0024244)

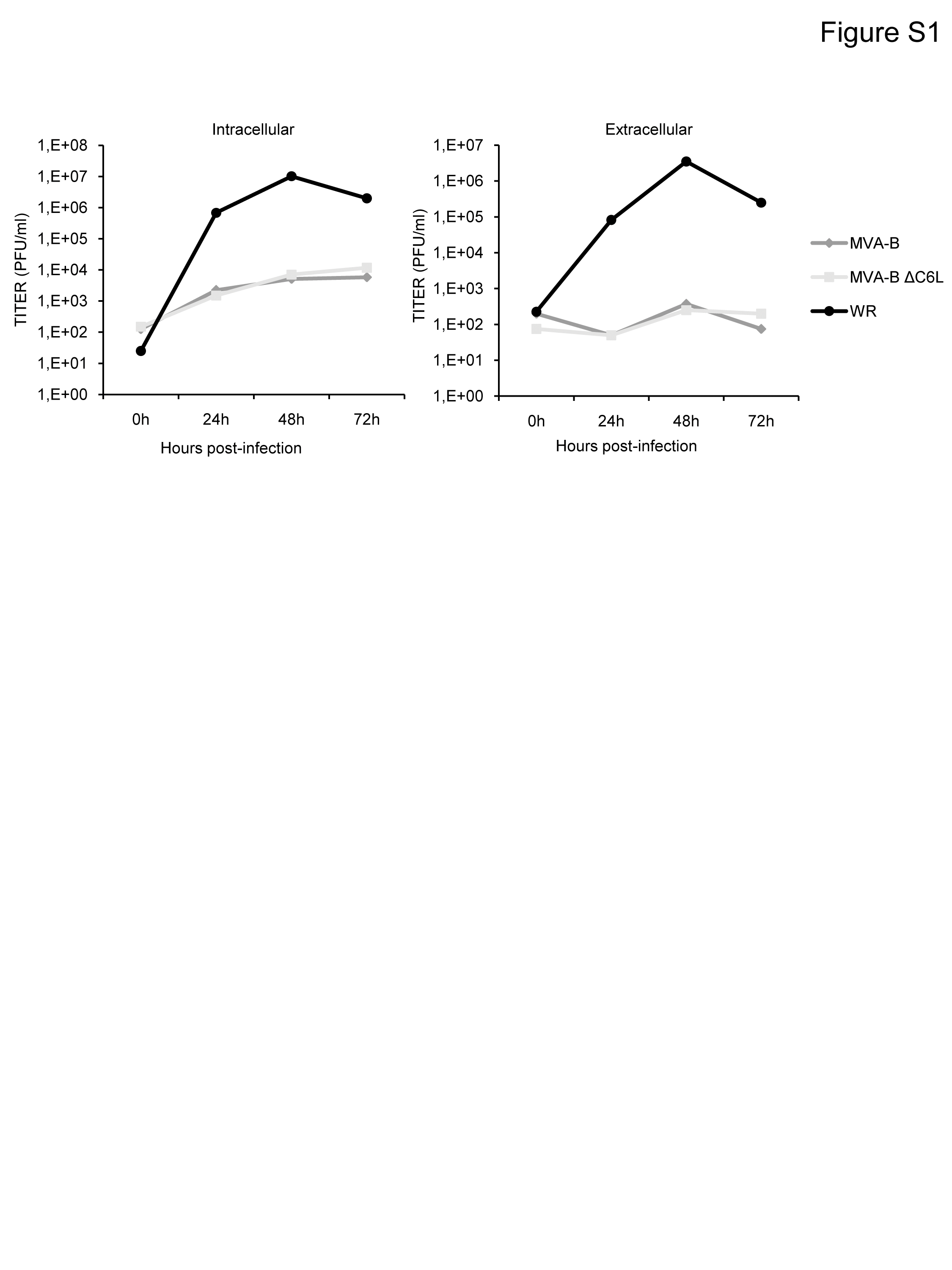

Supplement: Figure S1 — Virus growth of MVA-B and MVA-B ΔC6L in HeLa cells. Monolayers of HeLa cells were infected at 0.01 PFU/cell with WR, MVA-B or MVA-B ΔC6L for 0, 24, 48, and 72 h. For comparative purposes, we used the replication-competent WR strain. Cells were collected by centrifugation and infectious viruses associated with the cells (intracellular) and released to the medium (extracellular) during the course of the infection were measured by a plaque immunostaining assay with anti-WR antibodies. Data are from one experiment representative of two experiments. (TIF) [file pone.0024244.s001.tif]
